# The state of biosafety across China's CDC microbiology laboratories: insights from a nationwide survey (2021–2023)

**DOI:** 10.3389/fpubh.2024.1436503

**Published:** 2024-08-02

**Authors:** Peihua Niu, Zhenlu Sun, Ruiqing Zhang, Yiming Zhao, Fengyu Tian, Ping Cheng, Hongmei Zheng, Jianqiang Guo, Meng Zhang, Xuejun Ma, Ji Wang

**Affiliations:** ^1^NHC Key Laboratory of Medical Virology and Viral Diseases, National Institute for Viral Disease Control and Prevention, China Center for Disease Control and Prevention (CDC), Beijing, China; ^2^Yantai Center for Disease Control and Prevention, Yantai, China

**Keywords:** biosafety, CDC, Microbiology laboratories, China, nationwide survey

## Abstract

**Background:**

The COVID-19 pandemic underscored the critical importance of biosafety in microbiology laboratories worldwide. In response, China has ramped up its efforts to enhance biosafety measures within its Centers for Disease Control and Prevention (CDC) laboratories. This study provides the first comprehensive assessment of biosafety practices across provincial, city, and county levels of CDC microbiology laboratories in China.

**Methods:**

We conducted a nationwide cross-sectional survey from 2021 to 2023, targeting staff from microbiology laboratories within CDCs at all administrative levels in China. Stratified sampling was employed to select respondents, ensuring a representative mix across different CDC hierarchies, job titles, and academic qualifications. The survey encompassed questions on biosafety training, the presence of BSL-2 and BSL-3 laboratories, adherence to general biosafety guidelines, and management practices regarding specimens, reagents, and consumables. Statistical analysis was performed to identify significant differences in biosafety practices among different CDC levels.

**Results:**

A total of 990 valid responses were received, highlighting a nearly universal presence (98.69%) of BSL-2 laboratories and a significant yet varied presence of BSL-3 laboratories across the CDC network. The survey revealed high levels of biosafety training (98.69%) and adherence to biosafety protocols. However, challenges remain in the consistent application of certain safety practices, especially at lower administrative levels. Notable differences in the management of specimens, reagents, and consumables point to areas for improvement in ensuring biosecurity.

**Conclusion:**

Our findings indicate a robust foundation of biosafety practices within CDC microbiology laboratories in China, reflecting significant advancements in the wake of the Biosecurity Law's implementation. Nevertheless, the variability in adherence to specific protocols underscores the need for ongoing training, resources allocation, and policy refinement to enhance biosafety standards uniformly across all levels. This study's insights are crucial for guiding future improvements in laboratory biosafety, not just in China but potentially in other countries enhancing their public health infrastructures.

## 1 Background

The genesis and evolution of biosafety as a distinct and critical field of study embarked on its journey toward formalization and standardization in the United States during the mid-20th century (1, 2), leading to the formulation of expansive laboratory biosafety guidelines that continue to underpin contemporary practices (3, 4). In parallel, the People's Republic of China initiated the development of its legal and regulatory framework pertinent to biosafety and biosecurity in the 1980s (5). This endeavor gained considerable momentum in the early 21st century, reflecting a concerted effort to construct a robust biosafety infrastructure (6). A milestone in this developmental arc was the promulgation of the “Biosecurity Law of the People's Republic of China” in 2020, which became operative in 2021. This legislative enactment serves as a cornerstone, providing a robust legal structure to mitigate an array of biosafety challenges. Such legislative progress is anticipated to significantly bolster public health security, thereby amplifying the nation's capacity for the prevention and management of infectious diseases, marking a transformative phase in the augmentation of global biosafety norms.

China's public health architecture is characterized by a meticulously organized five-tier system, extending from the national echelon down through provincial, city, county, and township levels (7). At the heart of this network, the Centers for Disease Control and Prevention (CDCs), situated at provincial, city, and select county tiers, assume a pivotal role in the surveillance and management of infectious disease outbreaks, exemplified by the COVID-19 pandemic. Embedded within these CDCs are Microbiology Laboratories (Micro-Labs), entities dedicated to the identification and analysis of microbial and pathogenic entities. These labs are integral to the biosafety continuum, rendering the enhancement of personnel training and the vigilant monitoring of compliance with established biosafety protocols a paramount concern (8).

Cross-sectional surveys emerge as a potent tool for gauging the prevailing biosafety landscape within Micro-Labs, tailoring their focus to encompass a diverse array of research aims, including but not limited to biosafety training (9), incidences of biosafety accidents (10), and the biosecurity levels (11). Prior domestic inquiries have scrutinized biosafety awareness among 208 CDC Micro-Lab personnel spread over seven provinces, underlining the critical need for augmented training regimens (12). Despite these efforts, a discernible gap persists in the availability of an exhaustive, nationally encompassing, and longitudinally spanning survey dedicated to biosafety metrics.

To address this notable deficiency, we embarked on a comprehensive nationwide cross-sectional survey spanning the years 2021–2023, designed to delineate the biosafety posture of Micro-Labs situated within CDCs at the provincial, city, and county levels across China. This study specifically focuses on three main areas: General Biosafety Guidelines, Specimen Management Guidelines, and Reagent and Consumables Management Guidelines, among other aspects of biosafety. This extensive survey delved into various facets of biosafety, including the extent of biosafety training, the coverage of BSL-2 and BSL-3 laboratories, the coverage of protocols in general biosafety guidelines, and the management practices concerning specimens, reagents, and consumables. The findings from this study are instrumental in appraising the efficacy of extant biosafety implementations, pinpointing prevalent challenges in biosafety governance, and furnishing empirical data to inform and refine policy directives. Ultimately, this endeavor seeks to catalyze a substantive uplift in the biosafety benchmarks governing the CDC network, thereby fortifying the public health infrastructure against biosafety vulnerabilities.

## 2 Methods

### 2.1 Study participants and survey methodology

The study targeted staff from Micro-Labs within the CDCs at provincial, city, and county tiers throughout China, primarily engaged in pathogen detection and research activities. From 2021 to 2023, we employed a stratified sampling technique each year to select around 10 respondents in each province, aiming for a representative cross-section across diverse CDC hierarchies, job titles, academic qualifications, and roles. The validity and representativeness of this sampling approach were affirmed in a preliminary survey carried out in 2021 (13). Beyond basic demographic data, the questionnaire included five single-choice and four multiple-choice items, administered via a WeChat mini-program in June 2021, November 2022, and November 2023, with a 1-week window for response collection following each dissemination.

### 2.2 Statistical analysis approach

This study primarily quantifies the current state of biosafety by measuring the coverage rate of each protocol within the corresponding guidelines. For instance, “hand washing” is a protocol specified in the general biosafety guidelines, with its coverage rate calculated as the ratio of questionnaires marking “hand washing” to the total number of valid questionnaires. The Chi-square test is utilized to assess statistical differences in the coverage rates of protocols across provincial, city, and county levels (*P* < 0.05). When dealing with small sample sizes or low expected frequencies, the Fisher's exact test is applied to ensure precise probabilistic determination.

## 3 Results

### 3.1 Demographics

The survey garnered valid responses from 990 participants over 3 years. In 2021, there were 399 respondents (40.30%), 433 in 2022 (43.74%), and 158 in 2023 (15.96%). Among these, 67% were female, and 33% were male. The age distribution ranged from 16 to 58 years, primarily concentrated in the middle-age bracket, although the survey lacked age data for 2022 (mean age of 36.19 years, median age of 36). The distribution of CDC levels revealed 33% at the provincial level, 58% at the city level, and 9% at the county level. The highest participation rates were observed from Jiangxi (9.8%), Jiangsu (7.2%), and Guizhou (6.7%).

### 3.2 BSL-2 and BSL-3 laboratories

From 2021 to 2023, 98.69% (977 out of 990) of the respondents reported the presence of BSL-2 labs within their CDCs, with 98.15% at the provincial level, 99.13% at the city level, and 97.75% at the county level, showing no significant statistical differences between CDC levels or provinces. Additionally, 24.14% (239 out of 990) indicated the existence of BSL-3 labs, with a significant prevalence at the provincial level (62.96%) compared to 5.72% at city level and 2.25% at county level, indicating a higher probability of BSL-3 lab presence at provincial CDCs. Twenty-nine out of 31 provinces reported having BSL-3 labs.

### 3.3 General biosafety guidelines

**Figure 1:**
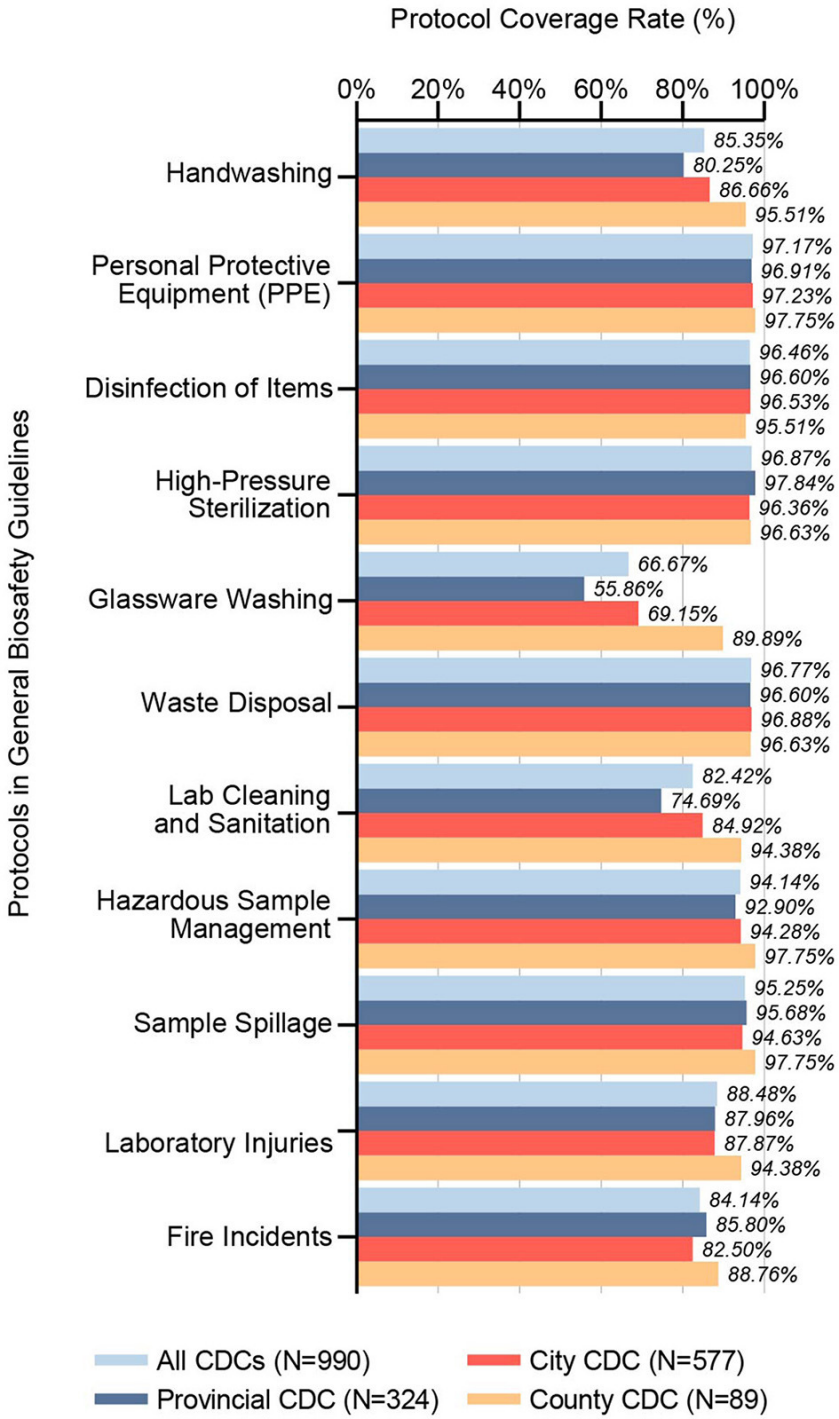
Coverage rates of protocols in general biosafety guidelines. This bar chart illustrates the inclusion rates of specific protocols within general biosafety guidelines across CDC microbiology laboratories in China, segmented by administrative divisions (Provincial CDC, City CDC, County CDC) and collectively for all CDCs surveyed (*N* = 990). It reveals the extent to which each protocol is represented in the guidelines, providing insight into the consistency and variations of biosafety protocol inclusion across the CDC network.

Between 2021 and 2023, 98.69% of respondents confirmed receiving biosafety training prior to commencing laboratory activities. A notable 99.29% of CDCs had established general biosafety guidelines, covering protocols on Personal Protective Equipment (PPE) (97.17%), Waste Disposal (96.77%), Disinfection of Items (96.46%), High-Pressure Sterilization (96.87%), Sample Spillage (95.25%), Hazardous Sample Management (94.14%), Laboratory Injuries (88.48%), Lab Cleaning and Sanitation (82.42%), Fire Incidents (84.14%), Hand Washing (85.35%), and Glassware Washing (66.67%), as shown in Figure 1. Statistical analysis revealed significant disparities across CDC tiers in the coverage rate of Hand Washing, Glassware Washing, and Lab Cleaning and Sanitation protocols.

### 3.4 Specimen management guidelines

**Figure 2:**
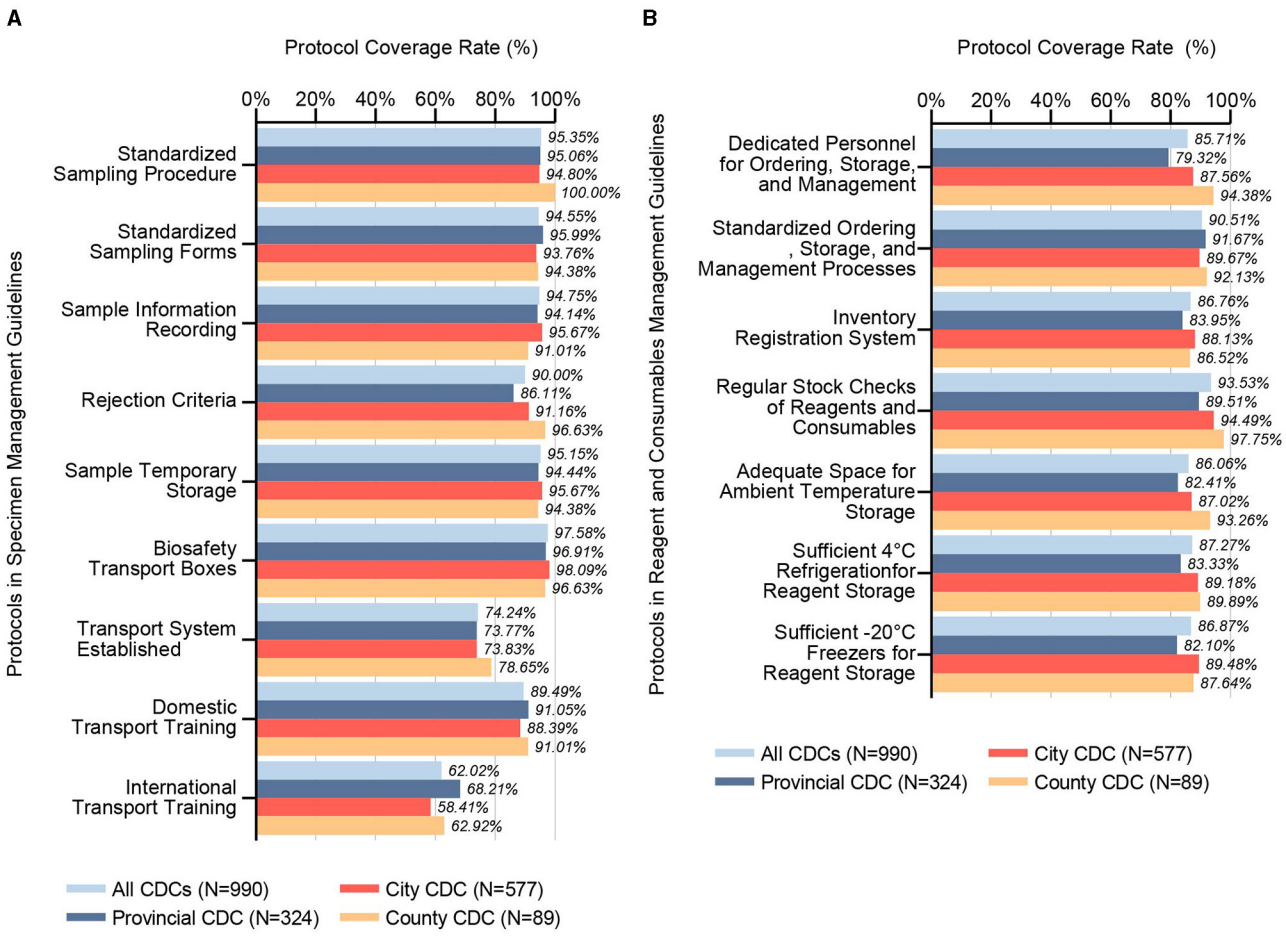
Coverage rates of specimen management guidelines and reagents and consumables management guidelines. **(A)** This bar chart details the inclusion rates of various protocols within specimen management guidelines across CDC microbiology laboratories, categorized by administrative divisions (Provincial, City, County) and cumulatively for all CDCs examined (*N* = 990). It assesses the presence and emphasis on key specimen management protocols, shedding light on the systematic approaches adopted across different tiers for handling and storing biological specimens, which is critical for maintaining biosafety and biosecurity. **(B)** Analogously, this chart reflects the inclusion rates for protocols within the reagents and consumables management guidelines. It scrutinizes practices such as stock management, storage conditions, and personnel responsibilities, highlighting the extent of adherence to best practices for managing essential laboratory inputs across various CDC levels. This comprehensive view not only underscores areas of uniformity in management practices but also points to potential gaps, informing strategies for enhancing the overall biosafety framework.

Our survey data from 2021 to 2023 delineated that specimen management guidelines across CDCs include protocols for Biosafety Transport Boxes (97.58%), Standardized Sampling Procedure (95.35%), Sample Temporary Storage (95.15%), Sample Information Recording (94.75%), Standardized Sampling Forms (94.55%), Rejection Criteria (90.00%), Domestic Transport Training (89.49%), Transport System Establishment (74.24%), and International Transport Training (62.02%), as shown in Figure 2A. Noteworthy, statistical differences were observed among CDC levels in coverage rate of Rejection Criteria and International Transport Training protocols.

### 3.5 Reagent and consumables management guidelines

The analysis for the period of 2021 to 2023 indicates that the management guidelines for reagents and consumables at CDCs incorporate protocols for Regular Stock Checks of Reagents and Consumables (93.53%), Standardized Ordering, Storage, and Management Processes (90.51%), Sufficient 4°C Refrigeration for Reagent Storage (87.27%), Sufficient−20°C Freezers for Reagent Storage (86.87%), Inventory Registration System (86.76%), Dedicated Personnel for Ordering, Storage, and Management (85.71%), and Adequate Space for Ambient Temperature Storage (86.06%), as shown in Figure 2B. Statistically significant differences were found between different levels of CDCs in five key areas: Dedicated Personnel, Regular Stock Checks, Adequate Space for Storage, and Sufficient Freezer Capacity for Reagent Storage.

### 3.6 Supply chain challenges

More than half of the respondents (54.44%) reported occasional issues with the supply chain, such as delivery delays, incorrect orders, and inappropriate transportation conditions for reagents and consumables. Meanwhile, 36.16% reported no such issues, 8.28% were unsure, and 1.11% frequently encountered these challenges. Additionally, the majority (81.21%) indicated their laboratories never use expired reagents, with 16.46% occasionally doing so, 2.02% unsure, and 0.3% often using expired items. A significant 97.68% of respondents affirmed never reusing disposable consumables, while a minority reported occasional (1.21%) or frequent (0.71%) reuse, and 0.4% were unsure.

### 3.7 Annual trends in biosafety practices and compliance

In addition to stratification by CDC levels, annual trends in biosafety practices were analyzed. No statistically significant differences were detected in the annual distribution of BSL-2 and BSL-3 laboratories, nor in the coverage rate of protocols within the General Biosafety Guidelines, Specimen Management Guidelines, Reagent and Consumables Management Guidelines, or in addressing Supply Chain Challenges. However, significant annual differences were observed in the use of expired reagents and the reuse of disposable consumables. Specifically, the percentage of respondents reporting never using expired reagents was 82.21% in 2021, 83.60% in 2022, and declined to 72.15% in 2023. Similarly, the percentage of those never reusing disposable consumables was 97.24% in 2021, increased to 98.61% in 2022, and slightly decreased to 96.20% in 2023.

## 4 Discussion

To the best of our knowledge, this investigation represents the most extensive biosafety survey within the realm of China's public health framework thus far. It encompasses an examination of 990 personnel engaged in microbiological endeavors within the CDCs at the provincial, city, and county levels across all 31 provincial administrative divisions. This ambitious study spanned from 2021 to 2023, thereby encompassing both the duration of the COVID-19 pandemic and the subsequent period. Our scrutiny was directed toward elements with a direct bearing on biosafety, notably the penetration of BSL-2 and BSL-3 laboratories, the incidence of biosafety training among staff, and the specifics outlined in the biosafety-related guidelines at their CDCs. Additionally, we delved into aspects with an indirect influence on biosafety, including the stewardship of specimens, reagents, and consumable materials, thereby providing a comprehensive overview of the biosafety landscape within these critical infrastructures.

Existing literature establishes that BSL-2 laboratories are adept at handling pathogens of significant risk, such as SARS-CoV-2 (14). This capability not only signifies the extensive acknowledgment and implementation of elementary biosafety measures across CDCs at diverse tiers and provinces but also substantially elevates the overall readiness and response efficiency to high-risk infectious diseases within the national framework. Furthermore, institutes operating at BSL-3 laboratories are critical for the investigation of pathogens classified within the third risk groups, encompassing basic, applied, and translational research domains. These higher-level laboratories necessitate advanced technological infrastructure and substantial financial investment for maintenance (15). Predominantly established within provincial-level CDCs, BSL-3 laboratories underscore the broader necessity for advanced research and robust response strategies against high-risk infectious agents, highlighting a strategic imperative at this administrative level. Despite the absence of reported excesses, vigilant oversight regarding the potential over-establishment of BSL-3 laboratories is advisable to circumvent the inefficient use of resources, particularly the concern over constructing redundant facilities within the same organization.

Prior studies have highlighted the pivotal role of biosafety training in elevating the comprehension and application of biosafety protocols among CDC laboratory staff, with recommendations extending to the inclusion of collegiate populations in such educational programs (16, 17). Our comprehensive examination reveals that almost all CDC laboratory personnel across the vast expanse of provinces and at varied hierarchical levels in China are recipients of rigorous biosafety training before their induction, guided by meticulously crafted biosafety guidelines. Our analysis discerns that the adherence to protocols, manifesting a coverage rate above 90%, is intricately linked to the direct manipulation of pathogens. In contrast, those falling below the 90% threshold predominantly address injury prevention and laboratory cleanliness, as shown in Figure 1. It is imperative to acknowledge the integral nature of laboratory operations, wherein every procedure is part of an interconnected web of activities, each bearing its inherent set of risks. Instances such as fire outbreaks potentially leading to sample spillage, the handling of waste material resulting in injuries, and the compromise of sterilization processes due to laboratory disarray exemplify the interlinked nature of laboratory safety concerns. Consequently, we call for a broadening of the focus within CDC biosafety guidelines to encapsulate an expansive array of safety and procedural protocols, thereby enhancing the comprehensive safety framework within these crucial public health institutions.

The PPE adherence in China's CDC microbiology laboratories, specified at 97.17% in guidelines, contrasts with actual implementation rates in global BSL-3/BSL-4 laboratories, which range from 54 to 90% as reported by survey data (18). This variation suggests that the enhanced biosafety protocols instituted during the COVID-19 pandemic may have contributed to more rigorous compliance in China. The pandemic has highlighted the critical need for robust biosafety measures and continuous training, providing valuable insights for improving laboratory safety standards on a global scale.

Analogous to the stipulations within biosafety guidelines, specimen management protocols distinctly prioritize operations directly associated with the secure manipulation and preservation of pathogenic specimens, pinpointing substantial vulnerabilities within the ambit of specimen transportation, as shown in Figure 2A. In contrast to sectors beyond the life sciences, biological specimens exhibit heightened sensitivity to environmental perturbations—including extremes of temperature, mechanical vibration, physical shock, and protracted transit durations—factors that critically endanger the specimen's structural and biosecurity integrity (19). Inadequate conditions during transportation and storage are implicated in the potential alteration of specimen-intrinsic microbial consortia or the destabilization of biomolecular markers (20–22). This underscores the imperative for meticulous scrutiny of the veracity inherent to contemporary omics-based research outputs, notably within the domains of microbiome and metabolome studies, thereby ensuring the reliability and applicability of these scientific advancements.

The logistics of transporting infectious specimens demand an integrated approach, engaging multiple governmental departments, including but not limited to healthcare, transportation, public security, and customs. The National Disease Control and Prevention Administration (NDCPA), vested with the requisite administrative capabilities, unlike the CDCs, is ideally positioned to spearhead initiatives aimed at facilitating seamless specimen transport across provincial boundaries. This leadership role entails guiding provincial and municipal NDCPAs in mirroring national protocols to orchestrate a comprehensive transportation framework within their respective domains. Such a coordinated endeavor is anticipated to lay the groundwork for a nationwide specimen transportation network characterized by its safety, legality, and efficiency. Amid the progressing tide of globalization and enhanced international cooperation in public health matters, the incremental adoption of validated specimen transportation schemas across nations presents a viable strategy, as evidenced by the efficacious transport logistics witnessed during the Ebola crisis (23, 24).

Within the CDC Micro-Labs, the stewardship of reagents and consumables exhibits commendable compliance, with coverage rate of established protocols surpassing the 85% threshold, as shown in Figure 2B. This efficacious management is ostensibly attributed to the CDCs' provision of sufficient financial and refrigeration resources designated for these materials (13). Nonetheless, the presence of adequate funding and storage facilities may inadvertently foster a propensity for overstocking, occasionally culminating in the utilization of expired materials. In 2023, a significant increase in the use of expired reagents and the recycling of disposable consumables, relative to previous years, may be attributed to comprehensive adjustments in China's COVID-19 prevention and control policies at the end of 2022. These adjustments potentially redefined operational objectives and altered the workload dynamics within CDC Micro-Labs, thereby intensifying fiscal pressures on health departments. Moreover, the conclusion of the pandemic likely precipitated structural transformations within the biopharmaceutical supply chain, with shifts in suppliers' business orientations leading to critical disruptions that adversely affected the supply of materials to Micro-Labs. Proactively designated procurement strategies and resource-sharing frameworks stand as viable preventive measures against such inefficiencies.

Notably, our inquiry reveals that a substantial proportion of participants acknowledge intermittent deficiencies within the supply chain of reagents and consumables. It is imperative that these discrepancies be promptly addressed within daily operational paradigms to avert the potential amplification of adverse outcomes amidst emergent infectious disease episodes. The advent of unforeseen supply chain interruptions, manifesting as delays in delivery and quantitative shortfalls, may necessitate the adoption of substandard materials by laboratory personnel. This includes, but is not limited to, the use of improperly sized PPE, pipette tips lacking filtration capabilities, or under-concentrated disinfectants, which collectively contribute to an escalation in biosafety vulnerabilities.

Within the CDC Micro-Labs, the stewardship of reagents and consumables exhibits commendable compliance, with adherence to established protocols surpassing the 85% threshold. This efficacious management is ostensibly attributed to the CDCs' provision of sufficient financial and refrigeration resources designated for these materials (13). Nonetheless, the presence of adequate funding and storage facilities may inadvertently foster a propensity for overstocking, occasionally culminating in the utilization of expired materials. Proactively designated procurement strategies and resource-sharing frameworks stand as viable preventive measures against such inefficiencies. Notably, our inquiry reveals that a substantial proportion of participants acknowledge intermittent deficiencies within the supply chain of reagents and consumables. It is imperative that these discrepancies be promptly addressed within daily operational paradigms to avert the potential amplification of adverse outcomes amidst emergent infectious disease episodes. The advent of unforeseen supply chain interruptions, manifesting as delays in delivery and quantitative shortfalls (25), may necessitate the adoption of substandard materials by laboratory personnel. This includes, but is not limited to, the use of improperly sized personal protective equipment, pipette tips lacking filtration capabilities, or under-concentrated disinfectants, which collectively contribute to an escalation in biosafety vulnerabilities.

Our investigation reveals a counterintuitive hierarchy in biosafety protocol adherence, with county-level CDCs demonstrating superior performance over city-level, and city-level surpassing provincial-level entities. This paradoxical observation diverges from the presumptive paradigm that higher echelons of CDCs would inherently maintain more stringent biosafety protocols. Our prior analysis in 2021, focusing on the operational competencies of CDC Micro-Labs, predominantly validated the expectation of ascending capabilities with rising organizational levels. Nonetheless, indications of a regression in basic experimental capacities were noted among top-tier CDCs, a phenomenon that may be ascribed to the differential operational mandates and strategic orientations across the CDC hierarchy (13). Irrespective of the delineated duties, the potential for biosafety incidents, along with their attendant risks and damages, remains a constant across all CDC levels. It is imperative for CDCs at higher levels to elevate biosafety to a paramount concern, paralleling their commitment to advanced laboratory proficiencies. This entails the expedited enhancement of biosafety guidelines coupled with the systematic execution of self-assessments, ensuring the perpetuation and advancement of biosafety standards across the spectrum of public health laboratories.

Following the promulgation and execution of the “Biosecurity Law of the People's Republic of China,” there has been a heightened focus on biosafety and biosecurity, establishing these domains as pivotal to the nation's strategic imperatives (26). Our survey elucidates existing deficiencies within biosafety and biosecurity frameworks, thereby aiding in the pursuit of biosafety management's sustainable implementation, with a particular emphasis on scenarios characterized by limited resources (27). Consequently, the detailed examination facilitated by our research, especially at the county-level CDCs, emerges as exceptionally consequential. The recent completion of the first National Field Epidemiology Survey Competition in 2024 highlighted the criticality of precise selection and procedural adherence in PPE utilization, emphasizing the necessity for ongoing biosafety education amongst all CDC professionals, extending beyond the confines of Micro-Labs. The Laboratory Quality Management System (LQMS) promotes the perpetual assessment and refinement of laboratory operations to preempt and ameliorate latent hazards (28). The recommended adoption of the LQMS framework by CDCs represents a forward-looking strategy to bolster biosafety measures. By embracing this blueprint, CDCs could establish a robust foundation for elevating laboratory biosafety management to a higher standard, ensuring a more comprehensive and systematic approach to biosafety across the public health spectrum.

## Data availability statement

The original contributions presented in the study are included in the article/Supplementary material, further inquiries can be directed to the corresponding authors.

## Ethics statement

Ethical approval was not required for the study involving humans in accordance with the local legislation and institutional requirements. The studies were conducted in accordance with the local legislation and institutional requirements. Written informed consent to participate in this study was not required from the participants in accordance with the national legislation and the institutional requirements.

## Author contributions

PN: Investigation, Methodology, Data curation, Writing – review & editing, Writing – original draft. ZS: Writing – original draft, Software, Formal analysis, Writing – review & editing, Data curation. RZ: Methodology, Writing – review & editing, Investigation. YZ: Investigation, Methodology, Writing – review & editing. FT: Investigation, Writing – review & editing, Methodology. PC: Investigation, Methodology, Writing – review & editing. HZ: Writing – review & editing, Funding acquisition. JG: Writing – review & editing, Funding acquisition. MZ: Funding acquisition, Writing – review & editing. XM: Writing – original draft, Supervision, Project administration, Data curation, Writing – review & editing. JW: Visualization, Writing – original draft, Data curation, Writing – review & editing, Methodology.
